# Roles and Clinical Significances of ATF6, EMC6, and APAF1 in Prognosis of Pancreatic Cancer

**DOI:** 10.3389/fgene.2021.730847

**Published:** 2022-02-11

**Authors:** Wang Xiao, Rong-Chang Cao, Wan-Jun Yang, Jie-Hui Tan, Ruo-Qi Liu, He-Ping Kan, Lei Zhou, Na Zhang, Zhi-Ye Chen, Xue-Mei Chen, Jia Xu, Guo-Wei Zhang, Peng Shen

**Affiliations:** ^1^ Division of Hepatobiliopancreatic Surgery, Department of General Surgery, Nanfang Hospital, Southern Medical University, Guangzhou, China; ^2^ Department of Hepoctobiliary Pancreatic Surgery, The Eighth Affiliated Hospital, Sun Yat-sen University, Shenzhen, China; ^3^ Department of Anesthesiology, Nanfang Hospital, Southern Medical University, Guangzhou, China; ^4^ Department of Occupational Health and Medicine, Guangdong Provincial Key Laboratory of Tropical Disease Research, School of Public Health, Southern Medical University, Guangzhou, China; ^5^ Department of Pathophysiology, Southern Medical University, Guangzhou, China; ^6^ Department of Oncology, Nanfang Hospital, Southern Medical University, Guangzhou, China

**Keywords:** pancreatic cancer, endoplasmic reticulum stress (ER stress), apoptosis, ATF6, EMC6, Apaf1, prognosis

## Abstract

**Background:** Pancreatic cancer (PC) is prevalent among malignant tumors with poor prognosis and lacks efficient therapeutic strategies. Endoplasmic reticulum (ER) stress and apoptosis are associated with chronic inflammation and cancer progression. However, the prognostic value of ER stress-related, and apoptosis-related genes in PC remains to be further elucidated. Our study aimed at confirming the prognostic values of the ER stress-related genes, ATF6, EMC6, XBP1, and CHOP, and the apoptosis-related gene, APAF1, in PC patients.

**Methods:** Gene Expression Profiling Interactive Analysis 2 (GEPIA2) was used to evaluate prognosis value of ATF6, EMC6, XBP1, CHOP, and APAF1 in PC. Clinical data from 69 PC patients were retrospectively analyzed. Immunohistochemistry, Western blotting, and qRT-PCR were used for the assessment of gene or protein expression. The cell counting kit-8 (CCK-8) and the Transwell invasion assays were, respectively, used for the assessment of the proliferative and invasive abilities of PC cells. The prognostic values of ATF6, XBP1, CHOP, EMC6, and APAF1 in PC patients were evaluated using Kaplan–Meier and Cox regression analyses.

**Results:** XBP1 and CHOP expressions were not associated with PC recurrence-free survival (RFS), overall survival (OS) and disease-specific survival (DSS). ATF6 upregulation and EMC6 and APAF1 downregulations significantly correlated with the poor RFS, OS, and DSS of PC patients. ATF6 promoted PC cell proliferation and invasion, while EMC6 and APAF1 inhibited these events.

**Conclusion:** ATF6 upregulation and EMC6 and APAF1 downregulations may be valid indicators of poor prognosis of PC patients. Moreover, ATF6, EMC6, and APAF1 may constitute potential therapeutic targets in PC patients.

## Introduction

Pancreatic cancer (PC) is the fourth most common cause of cancer-related death in the United States with a 5-years survival rate of 10% (Siegel et al., 2021). The main treatment options include surgery, chemotherapy, radiotherapy, targeted therapy, supportive care, and their combination, however, surgical resection is the only curative therapy (Mizrahi et al., 2020). However, post-surgical resection recurrence is observed in approximatively 80% of PC patients (Groot et al., 2019; Kim et al., 2019). Despite remarkable improvements in surgical techniques in recent years, surgically resected patients are susceptible to death from their disease due to the high rate of recurrence (Sakamoto et al., 2020). Thus, the evaluation of the prognosis of PC patients and the development of new therapeutic methods are urgently needed to improve PC prognostic efficiency.

The endoplasmic reticulum (ER) stress is induced by various physiological or pathological strains on the cell, such as glucose deprivation, hypoxia, or chemotherapeutics, that subsequently activate unfolded protein response (UPR) as an adaptive response for cell recovery from stress (Dauer et al., 2019). Protein kinase R-like ER kinase (PERK), the transcription factor 6 (ATF6), and the inositol requiring enzyme 1α (IRE1α) constitute the three branches of the UPR signaling pathway (Lin et al., 2019). X-box-binding protein 1 (XBP1) is generated through the activation of the IRE1α-mediated cleavage of XBP1 mRNA cleavage (Dauer et al., 2019; Barez et al., 2020), which expression is associated with poor prognosis in cancer patients (Bagratuni et al., 2010; Chen et al., 2014; Kwon et al., 2018). The C/EBP homologous protein (CHOP) is a downstream factor of severe ER stress (Cao et al., 2019), which is upregulated in response to dysregulated UPR and which is used in the stratification of mesothelioma patients (Dalton et al., 2013). ATF6 is a crucial regulator of the UPR pathway that is involved in coagulation (Zheng et al., 2019), and that has been identified as a poor prognosis factor in biliopancreatic carcinoma (Martinez-Useros et al., 2015) and colon cancer (Liu et al., 2018). Although ATF6, XBP1, and CHOP are involved in the prognosis of multiple diseases, their roles in PC remain not well-known.

ER membrane protein complex subunit 6 (EMC6) is a novel positive regulator of autophagy regulator in human cells (Shen et al., 2016) that has been demonstrated to influence the development of ER stress (Chitwood and Hegde, 2019), and to induce apoptosis in gastric cancer cells (Wang et al., 2017). Apoptotic protease-activating factor 1 (APAF1) is a crucial factor in the mitochondria-dependent death pathway, which also plays a significant role in ER stress-induced apoptosis (Shiraishi et al., 2006). In our previous study, we found that ATF6/XBP1/CHOP axis could promote the progression of chronic pancreatitis (CP) (Zhou et al., 2019), and EMC6 could upregulate the expression of APAF1 to promote pancreatic acinar apoptosis and inflammatory injury of CP (Tan et al., 2020). Given that CP was regarded as a high risk factor for PC, in the present study, we aimed at characterizing the expression of ER stress-related proteins ATF6/XBP1/CHOP/EMC6 and apoptosis-related protein APAF1 in PC, and at analyzing the relationship between their expression, the clinico-pathological variables, and prognosis of surgically resected PC patients.

## Materials and Methods

### Survival Analysis Based on Gene Expression Profiling Interactive Analysis 2

Different expressions of ATF6, EMC6, XBP1, CHOP, and APAF1 in PC and normal tissues were analyzed in Gene Expression Profiling Interactive Analysis 2 (GEPIA2; http://gepia2.cancer-pku.cn/#index). GEPIA2 is an interactive web server for analyzing the expression data of RNA from 9,736 tumors and 8,587 normal samples from the Cancer Genome Atlas (TCGA) and Genotype-Tissue Expression (GTEx) datasets (Tang et al., 2019). The *p*-value cutoff of the expression of gene for analysis was 0.01. The |Log2FC| cutoff of the expression of gene for analysis was 1, and we used log2 (TPM + 1) for log scale.

Survival analysis for overall survival (OS) and disease-free survival (DFS) in GEPIA2 was also used to estimate the relationship between PC prognostic value and the expression of genes ATF6, EMC6, XBP1, CHOP, and APAF1. The meaning of DFS was similar to recurrence-free survival (RFS) for this study. Hazards ratio (HR) was calculated based on Cox PH Model. The 95% confidence interval (CI) was shown by dotted line. The expression median value of gene for analyzing was identified as group cutoff to distinguish the high-expression group and the low-expression group.

### Patients and Tissue Samples

PC and adjacent normal pancreatic tissue samples were retrospectively collected from 69 PC patients, including 39 males and 30 females, with a mean age of about 57 years ranging from 34 to 79 years. These patients underwent surgical resections in Nanfang Hospital, Southern Medical University, between October 2010 and April 2019. A more detailed information about the patients is shown in Supplementary Table S1. Before the experiments, each patient provided a written informed consent. After resection, each sample was frozen at −80°C until analysis and all patients were followed up until May 2019. Complete clinical and pathological data and follow-up documentations were recorded and analyzed for all patients in the study, who had never received preoperative chemotherapy or radiotherapy. The tumor stages in the study were classified according to the Union for International Cancer Control (UICC). RFS was defined as the period from the date of pancreatic resection until the date of recurrence diagnosis. OS was defined as the period from the date of surgical resection until the date of death or last follow-up. Disease-specific survival (DSS) was defined as the period from the date of surgical resection until the date of death due to PC. The collection and analysis of tissue and data were approved by the Ethics Committee of the Southern Medical University.

### Hematoxylin and Eosin Staining and Immunohistochemistry

Pancreatic tissues were fixed in 4% neutral phosphate-buffered formalin, embedded in paraffin, and cut into 5-μm thick sections. Hematoxylin and eosin (H&E) staining were performed by experienced pathologists and followed by double-blinded histological evaluations. For the immunohistochemical detection, the sections were sequentially incubated overnight at 4°C with anti-ATF6 (Bioss, diluted 1:100), anti-XBP1 (Bioss, diluted 1:200), anti-CHOP (Bioss, diluted 1:100), anti-EMC6 (Proteintech, diluted 1:100) and anti-APAF1 (Abcam, diluted 1:100) antibodies. After 30 min of incubation with secondary antibodies at room temperature, the sections were counterstained with DAB solution and hematoxylin. Positively stained cells were evaluated by two experienced pathologists according to a previously described protocol (Zhou et al., 2020). Five high magnification areas were evaluated from each sample.

The immunohistochemical scores were assessed based on the intensity of staining and the proportion of stained cells. The scores of 0, 1, 2, and 3, respectively, corresponded to negative, weak, moderate, and strong staining intensities. The proportion of positively stained cells for each intensity was scored as follows: 0 (0%–5% positive cells), 1 (5%–25% positive cells), 2 (26%–50% positive cells), 3 (51%–75% positive cells), and 4 (76%–100% positive cells). The IHC scores given by each pathologist were calculated by multiplying the proportion of positively stained cells by the staining intensity scores. The final IHC scores were the mean value of scores from two pathologists and divided into low expression (0–7) and high expression (8–12) groups.

### Cell Culture and Transfection

The human PC cell lines, SW1990, HUPT4, PATU8988, PANC1, and ASPC1, were obtained from American Type Culture Collection (ATCC, Rockville, MD, USA). All the cell lines were cultured in Dulbecco’s modified Eagle’s medium (DMEM, Gibco). All mediums were supplemented with 10% fetal bovine serum (FBS) and maintained in a 37°C and 5% CO_2_ atmosphere.

ATF6, EMC6, and APAF1 expression and functions were investigated by Western blotting, qRT-PCR, CCK8 assay, and Transwell assay. Si-ATF6, Si-EMC6, and Si-APAF1 were, respectively, used to inhibit the expression of ATF6, EMC6, and APAF1. The constructs OE-ATF6, OE-EMC6, and OE-APAF1 were used to, respectively, overexpress ATF6, EMC6, and APAF1. The negative control (NC) and the vector were designed and synthesized by RiboBio Co., Ltd. (Guangzhou, China). PC cells were separately seeded in 24-well plates at a density of 5 × 10^5^ cells and transfected with Si-ATF6, Si-EMC6, Si-APAF1, OE-ATF6, OE-EMC6, OE-APAF1, NC, and the vector using riboFECT mRNA Transfection Reagent (RiboBio Co., Ltd. Guangzhou, China) according to the recommendations of the manufacturer. After 48 h of transfection and incubation in a 37°C and 5% CO_2_ atmosphere, the cells were harvested for subsequent experiments.

### Quantitative Real-Time PCR

Trizol Reagent (Merck, Germany) was used to extract RNA from cultured cells according to the instructions of the manufacturer. QRT-PCR was performed using the SYBR Premix Ex Tag kit (Takara Biotechnology Co., Ltd.) and the Applied Biosystems 7500 Real-Time PCR system (Thermo Fisher Scientific Inc., UK). The primers were designed and synthesized by RiboBio Co., Ltd. (Guangzhou, China). ATF6 forward, 5′-CGC CTT TTA GTC CGG TTC TT-3′ and reverse, 5′-CCA GTT GGT AAC AAT GCC ATG T-3′; EMC6 forward, 5′-GTC GCC AAG ATT TGC TCC CT-3′ and reverse, 5′-AAA CAC ACA ATG CCG GTA CAC-3′; APAF1 forward, 5′-GAT CCA CAC AGG CCA TCA CA-3′ and reverse, 5′-GGC GGG AGT CTA TGT TCC AC-3′. GAPDH forward, 5′-ATC ATC AGC AAT GCC TCC TG-3′ and reverse, 5′-ATG GAC TGT GGT CAT GAG TC-3′. ATF6, EMC6, and APAF1 expressions were normalized by GAPDH. The 2−^△△Ct^ method was used to calculate the relative expression levels. The expression levels of the genes were measured by qRT-PCR.

### Western Blot Assay

RiPA buffer (GenStar, China) was used to extract the proteins from the transfected cell lines. The proteins were loaded and separated on SDS-PAGE, and transferred onto PVDF membranes (Millipore, USA). After blocking with 5% non-fat milk, the membranes were incubated with anti-ATF6 (Bioss, China), anti-EMC6 (Proteintech, USA), anti-APAF1 (Abcam, UK), and anti-GAPDH (Fude Biological Technology Co., Ltd. China) antibodies overnight at 4°C. Following this step, the membranes were incubated with horseradish peroxidase-coupled secondary antibodies for 1 h, and finally, the expression of the proteins was revealed using the enhanced chemiluminescence solution (ECL, PerkinElmer, USA).

### Cell Counting Kit-8 Assay

The cell counting kit-8 assay was performed to assess the viability of the transfected cell lines. The transfected cells were seeded in a 96-well plate and cultured for 72 h. Then, 10 μl of CCK8 solution (Dojindo, Japan) was added to each well and incubated for 2 h. The absorbance at 450 nm was measured using a microplate reader.

### Transwell Invasion Assay

The Transwell invasion assay was performed to measure the invasion ability of the transfected cells using the Transwell chambers. The transfected cells were suspended in serum-free medium and seeded in the upper chambers that were precoated with Matrigel. The medium containing 10% FBS was placed in the lower chamber. After 24 h of incubation at 37°C, the cells on the bottom chamber were stained with 0.1% crystal violet for 10 min at 37°C. The number of stained cells in the lower chambers was calculated using a microscope.

### Statistical Analysis

The SPSS software version 26.0 (SPSS, Chicago, IL) and the GraphPad Prism software version 8.2 (San Diego, CA, USA) were used for statistical analyses. The data were reported as mean ± standard deviation. The paired Student’s t-test or the chi-square test were used to analyze the expression differences of genes among normal, high, and low expression groups. The Kaplan–Meier method was performed for survival curves between low-expression and high-expression groups using the log-rank test. To identify the factors involved in PC, the Cox proportional hazards regression method was applied using univariate and multivariate analyses. Differences were considered significant if *p* < 0.05.

## Results

### Elevated Expression of ATF6 and Reduced Expression of EMC6 and APAF1 Associated with Worse Prognosis in PC According to the GEPIA2 Database

**FIGURE 1:**
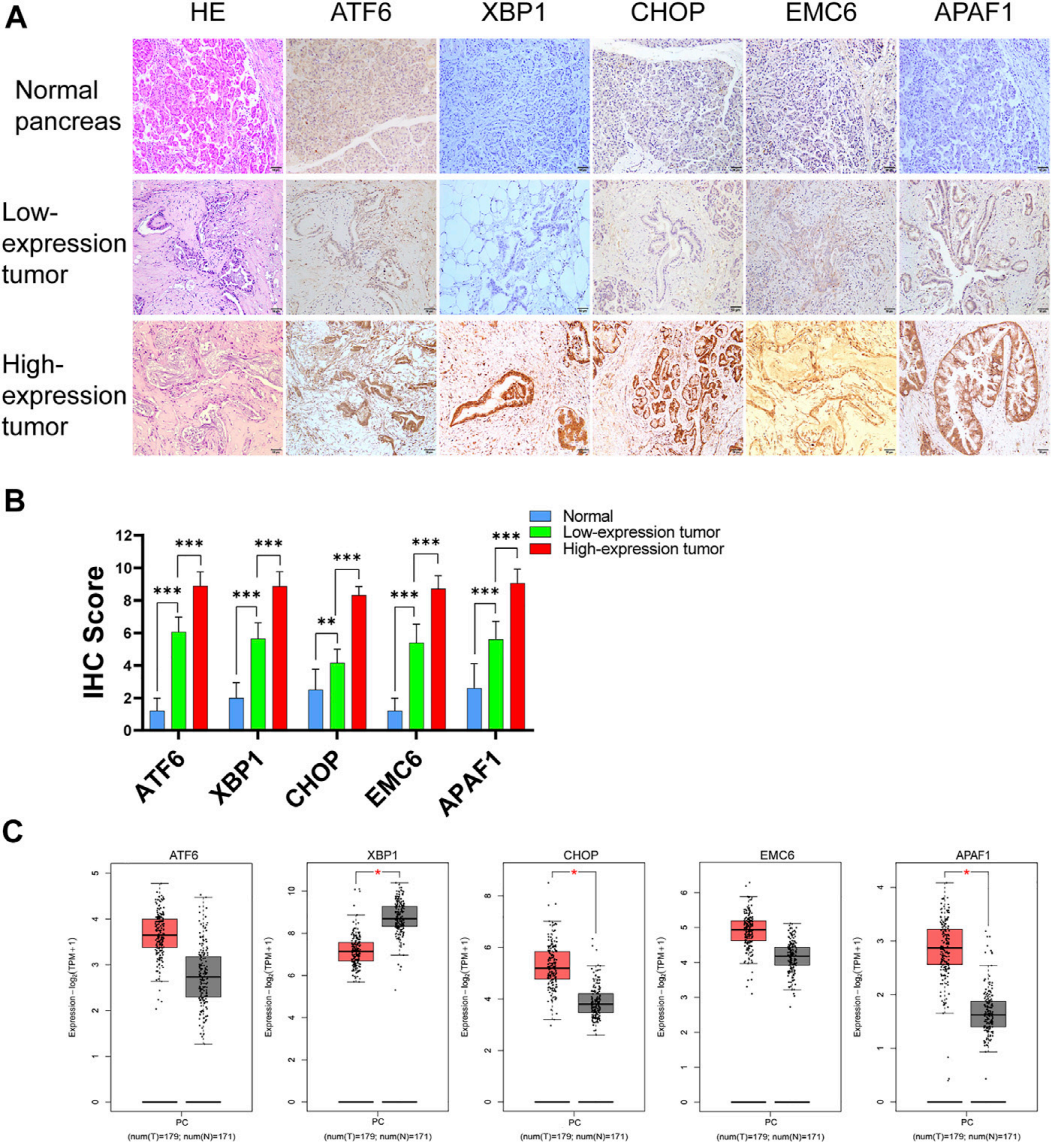
Analysis of ATF6, XBP1, CHOP, EMC6, and APAF1 expression in tumor and adjacent normal tissue samples of human. **(A)** H&E staining and immunohistochemical detection of ATF6, XBP1, CHOP, EMC6, and APAF1 protein expression in pancreatic tissue from normal and PC patients. **(B)** The expression of ATF6, XBP1, CHOP, EMC6, and APAF1 in tumor and normal samples. **(C)** The expression of ATF6, XBP1, CHOP, EMC6, and APAF1 in tumor (red) and normal samples (gray) *via* GEPIA2. T, tumors; N, normal tissues; *p ≤ 0.05, **p ≤ 0.01, ***p ≤ 0.001. Scale bars = 50 μm. ATF6, transcription factor 6; XBP1, X-box-binding protein 1; CHOP, C/EBP homologous protein; EMC6, ER membrane protein complex subunit 6; APAF1, apoptotic protease-activating factor 1; H&E, hematoxylin and eosin; PC, pancreatic cancer; GEPIA2, gene expression profiling interactive analysis.

**FIGURE 2:**
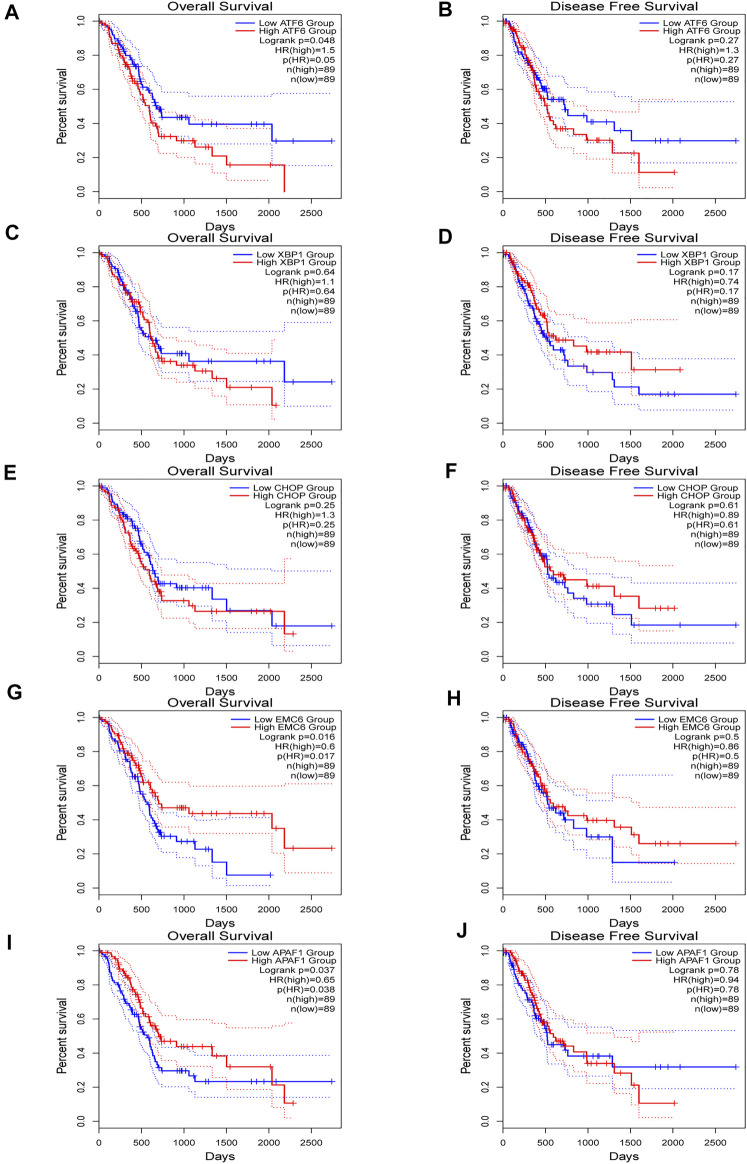
Kaplan–Meier plotter for overall survival (OS) **(A,C,E,G,I)** and disease-free survival (DFS) **(B,D,F,H,J)** based ATF6, XBP1, CHOP, EMC6, or APAF1 expression *via* survival analysis in GEPIA2 database. The two-sided log-rank test was performed to compare differences by *p*-values.

GEPIA2 was used to evaluate the relationship between the expression of ATF6, EMC6, XBP1, CHOP, APAF1, and prognosis value of PC. There was an upregulated trend of ATF6, EMC6, APAF1, and CHOP expression in PC compared with that in normal pancreatic tissues, while the result of XBP1 was opposite in this event (Figure 1C). Noticeably, the elevated expression of ATF6 and reduced expression of EMC6 and APAF1 showed a statistically significant association with poor OS for PC, but not with DFS, while the expression level of CHOP and XBP1 had no correlation with OS and DFS of PC (Figure 2).

### Clinicopathological Characteristics of Patients With Pancreatic Cancer

The clinicopathological features of the 69 patients are described in Table 1. Briefly, the median age of the patients at diagnosis was 58 years (age range: 34–79 years), and 39.1% of patients were >60 years old and 63.8% had tumors with sizes>3 cm. Patients were male in 56.5%, and the proportion of smokers was 23.2%. Adenocarcinoma was the most common histological type that was observed in 95.7% of patients. Stage I (42.0%) and II (44.9%) were common, with involvement of lymph nodes, and vascular and neural invasions observed in 31.9%, 13.0%, and 26.1%, respectively.

**TABLE 1:** Clinicopathological characteristics of patients with pancreatic cancer (PC).

Variable	Number (%)
Age (years)	
≤60	42 (60.9%)
>60	27 (39.1%)
Gender	
Male	39 (56.5%)
Female	30 (43.5%)
Smoking	
Yes	16 (23.2%)
No	53 (76.8%)
Tumor size	
≤3 cm	25 (36.2%)
>3 cm	44 (63.8%)
Histology	
Adenocarcinoma	66 (95.7%)
Others	3 (4.3%)
Stage	
I	29 (42.0%)
II	31 (44.9%)
III	2 (2.9%)
IV	7 (10.1%)
Lymph nodes involved	
Yes	22 (31.9%)
No	47 (68.1%)
Vascular invasion	
Yes	9 (13.0%)
No	60 (87.0%)
Neural invasion	
Yes	18 (26.1%)
Noo	51 (73.9%)

### Elevated Expression of Endoplasmic Reticulum Stress and Apoptosis-Related Proteins in Pancreatic Cancer

To determine the expression levels of ER stress-related and apoptosis-related proteins in human PC, we collected PC and normal pancreatic tissues for immunohistochemistry analysis. The expression of the ER stress-related proteins, ATF6, XBP1, CHOP, and EMC6, and the apoptosis-related protein, APAF1, were significantly higher in PC tissues compared with those in normal pancreatic tissues (Figures 1A,B). Compared with these results to GEPIA2, the reason for the different results in XBP1 may be the differences of sources of data analyzed. The RNA-seq datasets were used by GEPIA2, instead of the IHC data. These results demonstrate that the expressions of ATF6, XBP1, CHOP, EMC6, and APAF1 were upregulated in PC.

### Prognostic Values of Transcription Factor 6, ER Membrane Protein Complex Subunit 6, and Apoptotic Protease-Activating Factor 1 in Pancreatic Cancer Patients

**FIGURE 3:**
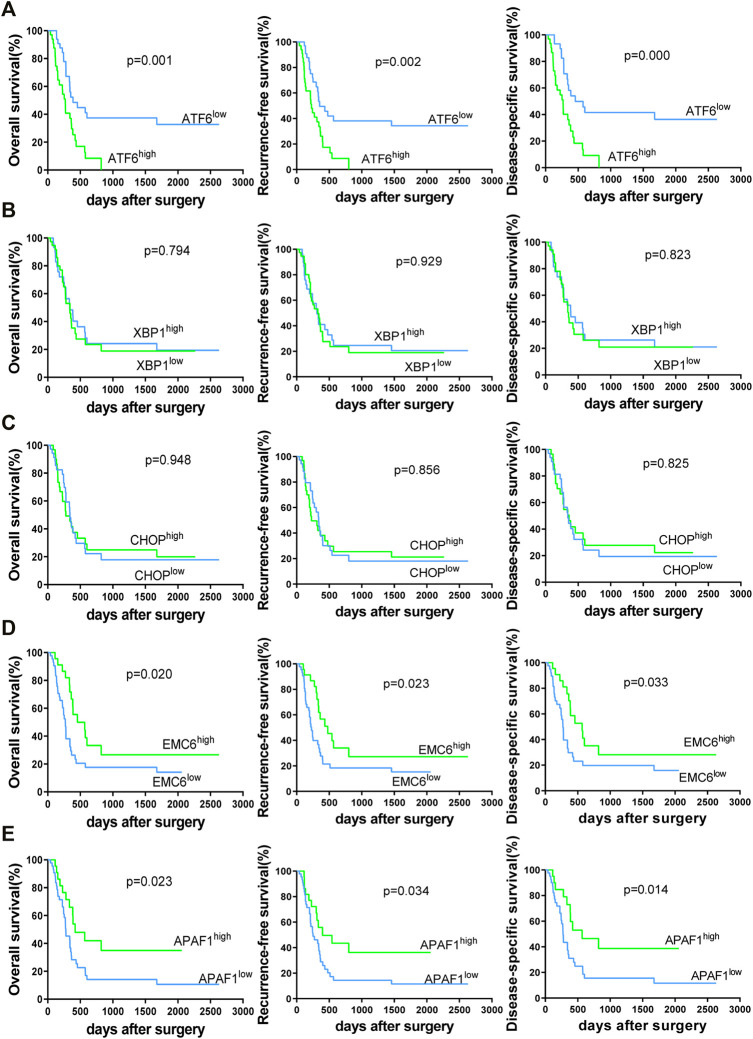
Kaplan–Meier survival curves of 69 PC patients for overall survival (OS), recurrence-free survival (RFS) and disease-specific survival (DSS)-based ATF6 **(A)**, XBP1 **(B)**, CHOP **(C)**, EMC6 **(D)**, or APAF1 **(E)** expression. The two-sided log-rank test was performed to compare differences by *p*-values.

To validate the associations between clinicopathological and molecular variables, and prognostic values in the PC patients, survival analysis was performed using the Kaplan–Meier method and the significance was tested with the log rank test. Univariate Cox regression analysis revealed that the TNM stage, lymph node involvement, and the expression levels of ATF6, EMC6, and APAF1, significantly correlate with RFS, OS, and DSS, while no significant differences were observed between the two groups based on age, sex, smoking, tumor size, neural and vascular invasions, and XBP1 and CHOP expression (Tables 2–4). Notably, the multivariate Cox regression analysis revealed that the TNM stage [HR = 3.578; *p* = 0.027], ATF6 expression [HR = 0.220; *p* < 0.001], EMC6 expression [HR = 2.571; *p* = 0.020], and APAF1 expression [HR = 2.426; *p* = 0.026] were also independent prognostic factors for RFS (Table 2). Simultaneously, the TNM stage [HR = 4.064; *p* = 0.014], lymph node involvement [HR = 0.380; *p* = 0.034], ATF6 expression [HR = 0.229; *p* = 0.001], EMC6 expression [HR = 2.956; *p* = 0.010], and APAF1 expression [HR = 2.369; *p* = 0.034] were also found to be significant prognostic factors for OS (Table 3). Moreover, the TNM stage [HR = 4.073; *p* = 0.017], lymph node involvement [HR = 0.396; *p* = 0.046], ATF6 expression [HR = 0.183; *p* < 0.001], EMC6 expression [HR = 3.275; *p* = 0.015], and APAF1 expression [HR = 2.887; *p* = 0.029] were also prognostic factors for DSS (Table 4). Kaplan–Meier survival plots indicated significantly higher survival rates at each time point for the ATF6^low^, EMC6^high^, and APAF1^high^ groups compared with those in the ATF6^high^, EMC6^low^ and APAF1^low^ groups (*p* < 0.05, Figures 3A,D,E). There was no statistical significance for XBP1 and CHOP (*p* > 0.05, Figures 3B,C). Discrepancy of results in ATF6, EMC6, and APAF1 from GEPIA2 survival analysis and our experiment may be due to different sources of data analyzed. Another reason probably lies in the different group cutoff in distinguishing the high-expression group and the low-expression group. The samples were divided into two groups by expression median of genes in GEPIA2 database, instead of defining “high expression” as the IHC scores ≥8 in our study. Nevertheless, both GEPIA2 survival analysis and our study identified that the expression of ATF6, EMC6, and APAF1 was related to PC patients’ survival. The results demonstrate that among the genes that are related to ER stress and apoptosis in this research, only ATF6, EMC6, and APAF1 were associated with PC patients’ survival, which would be used in further studies.

**TABLE 2:** Univariate and multivariate Cox regression analysis for recurrence-free survival.

Variable	Univariate analysis		Multivariate analysis	
	HR (95% CI)	*p*-Value	HR (95% CI)	*p*-Value
Age (≤60 vs. >60 years)	1.751 (0.964–3.180)	0.066		
Gender (male vs. female)	0.833 (0.464–1.496)	0.541		
Smoking (yes vs. no)	1.184 (0.571–2.457)	0.650		
Size (>3 vs. ≤3 cm)	0.581 (0.301–1.124)	0.107		
Stage (I, II, III vs. IV)	5.109 (2.121–12.305)	0.000^a^	3.578 (1.154–11.099)	0.027^a^
Lymph nodes involved (yes vs. no)	0.435 (0.240–0.788)	0.006^a^		
Neural invasion (yes vs. no)	1.044 (0.484–2.253)	0.912		
Vascular invasion (yes vs. no)	0.572 (0.264–1.240)	0.157		
ATF6 (high vs. low)	0.392 (0.213–0.720)	0.003^a^	0.220 (0.095–0.510)	0.000^a^
XBP1 (high vs. low)	1.027 (0.573–1.840)	0.929		
CHOP (high vs. low)	0.948 (0.530–1.694)	0.856		
EMC6 (high vs. low)	2.056 (1.091–3.874)	0.026^a^	2.571 (1.160–5.700)	0.020^a^
APAF1 (high vs. low)	2.017 (1.040–3.911)	0.038^a^	2.426 (1.114–5.281)	0.026^a^

Note. HR, hazard ratio.

95% CI, 95% confidence interval.

a Statistically significant results (*p* < 0.05).

**TABLE 3:** Univariate and multivariate Cox regression analysis for overall survival.

Variable	Univariate analysis		Multivariate analysis	
	HR (95% CI)	*p*-Value	HR (95% CI)	*p*-Value
Age (≤60 vs. >60 years)	1.880 (1.034–3.419)	0.039^a^		
Gender (male vs. female)	0.823 (0.458–1.478)	0.514		
Smoking (yes vs. no)	1.137 (0.547–2.362)	0.731		
Size (>3 vs. ≤3 cm)	0.599 (0.310–1.158)	0.128		
Stage (I, II, III vs. IV)	6.648 (2.653–16.657)	0.000^a^	4.064 (1.325–12.465)	0.014^a^
Lymph nodes involved (yes vs. no)	0.426 (0.236–0.769)	0.005^a^	0.380 (0.155–0.932)	0.034^a^
Neural invasion (yes vs. no)	0.958 (0.422–2.074)	0.913		
Vascular invasion (yes vs. no)	0.545 (0.252–1.181)	0.124		
ATF6 (high vs. low)	0.378 (0.206–0.696)	0.002^a^	0.229 (0.099–0.530)	0.001^a^
XBP1 (high vs. low)	1.080 (0.603–1.936)	0.795		
CHOP (high vs. low)	0.981 (0.549–1.753)	0.948		
EMC6 (high vs. low)	2.082 (1.105–3.924)	0.023^a^	2.956 (1.290–6.772)	0.010^a^
APAF1 (high vs. low)	2.117 (1.092–4.103)	0.026^a^	2.369 (1.069–5.249)	0.034^a^

Note. HR, hazard ratio.

95% CI, 95% confidence interval.

a Statistically significant results (*p* < 0.05).

**TABLE 4:** Univariate and multivariate Cox regression analysis for disease-specific survival.

Variable	Univariate analysis		Multivariate analysis	
	HR (95% CI)	*p*-Value	HR (95% CI)	*p*-Value
Age (≤60 vs. >60 years)	2.029 (1.077–3.823)	0.029^a^		
Gender (male vs. female)	0.865 (0.467–1.602)	0.645		
Smoking (yes vs. no)	1.068 (0.508–2.245)	0.861		
Size (>3 vs. ≤3 cm)	0.592 (0.296–1.184)	0.138		
Stage (I, II, III vs. IV)	7.494 (2.865–19.604)	0.000^a^	4.073 (1.284–12.922)	0.017^a^
Lymph nodes involved (yes vs. no)	0.396 (0.213–0.739)	0.004^a^	0.396 (0.159–0.983)	0.046^a^
Neural invasion (yes vs. no)	1.016 (0.445–2.319)	0.969		
Vascular invasion (yes vs. no)	0.546 (0.239–1.246)	0.150		
ATF6 (high vs. low)	0.327 (0.170–0.631)	0.001^a^	0.183 (0.077–0.438)	0.000^a^
XBP1 (high vs. low)	1.073 (0.579–1.988)	0.824		
CHOP (high vs. low)	1.072 (0.579–1.983)	0.826		
EMC6 (high vs. low)	2.028 (1.044–3.937)	0.037^a^	3.275 (1.255–8.550)	0.015^a^
APAF1 (high vs. low)	2.397 (1.170–4.909)	0.017^a^	2.887 (1.112–7.496)	0.029^a^

Note. HR, hazard ratio.

95% CI, 95% confidence interval.

a Statistically significant results (*p* < 0.05).

### The Expression of Transcription Factor 6, ER Membrane Protein Complex Subunit 6, and Apoptotic Protease-Activating Factor 1 in Pancreatic Cancer Cell Lines

**FIGURE 4:**
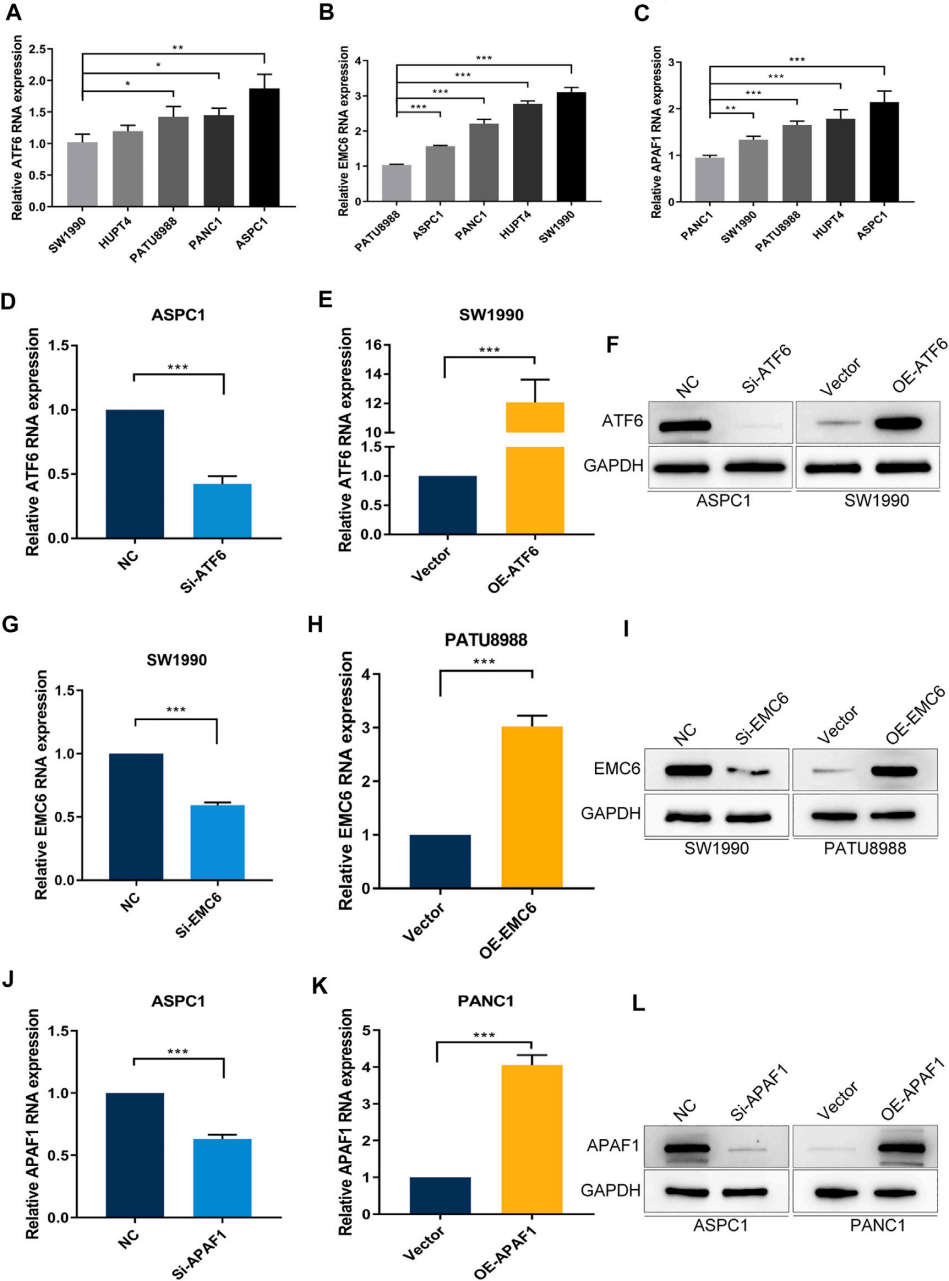
PC cell lines with different expression levels of ATF6, EMC6, and APAF1. The expression of **(A)** ATF6, **(B)** EMC6, and **(C)** APAF1 in different PC cell lines were detected by qRT-PCR. ATF6 expression in ASPC1 and SW1990 cell lines that were transfected with Si-ATF6 and OE-ATF6, respectively, were detected by qRT-PCR **(D,E)** and Western blot **(F)**. The expression of EMC6 in SW1990 and PATU8988 cell lines that were transfected with Si-EMC6 and OE-EMC6, respectively, were measured by qRT-PCR **(G,H)** and Western blot **(I)**. APAF1 expression in ASPC1 and PANC1 cell lines that were transfected with Si-APAF1 and OE-APAF1, respectively, were evaluated by qRT-PCR **(J,K)** and Western blot **(L)**. *p ≤ 0.05, **p ≤ 0.01, ***p ≤ 0.001.

To evaluate the expression of ATF6, EMC6, and APAF1 in pancreatic carcinoma cells by qRT-PCR, the PC cell lines, SW1990, HUPT4, PATU8988, PANC1, and ASPC1, were used. Noticeably, the highest and the lowest level of expression of ATF6 was observed in the cell lines, ASPC1 and SW1990 (Figure 4A). Therefore, SW1990 and ASPC1 were used as representative PC cell lines for ATF6 subsequent studies. Similarly, PATU8988 and SW1990 were selected for EMC6, PANC1, and ASPC1 was selected for APAF1 experiments (Figures 4B,C).

### High Expression of Transcription Factor 6 and Low Expression of ER Membrane Protein Complex Subunit 6, or Apoptotic Protease-Activating Factor 1 Promote Proliferative and Invasive Abilities of Pancreatic Cancer Cells

To further explore the function of ATF6, EMC6, and APAF1 in PC, we determined the effect of ATF6, EMC6, APAF1 on the proliferation and invasion abilities of PC cells using the CCK8 and Transwell assays on ATF6 transfected PC cell lines, SW1990 and ASPC1, EMC6 transfected PC cell lines, PATU8988 and SW 1990, APAF1 transfected cell lines, PANC1 and ASPC1. For this, qRT-PCR and Western blotting were used and revealed that the expression of ATF6, EMC6, and APAF1 were markedly increased in PC cells that were transfected with OE-ATF6, EMC6, and APAF1, and markedly inhibited in PC cells transfected with Si-ATF6, EMC6, and APAF1 when compared with the control (Figures 4D–L).

**FIGURE 5:**
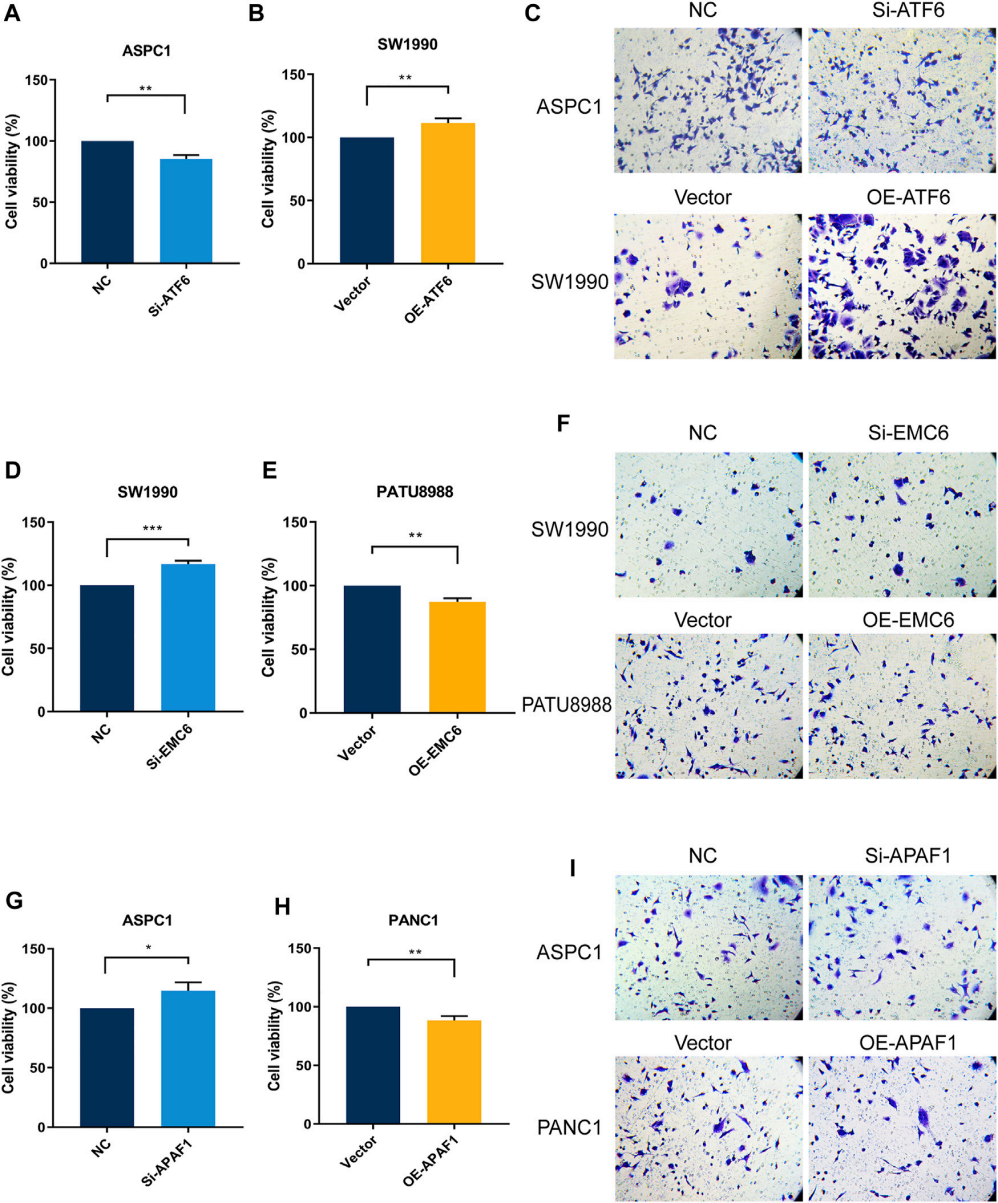
ATF6 promoted PC cell viability and invasion, while EMC6 and APAF1 inhibited these events. ATF6 effects on cell viability and invasion of Si-ATF6 transfected ASPC1 cells and OE-ATF6 transfected SW1990 cells were determined using **(A,B)** Cell-counting kit-8 (CCK-8) assay and **(C)** Transwell assay, respectively. **(D,E)** CCK8 and **(F)** Transwell assays were, respectively, performed to measure cell viability and invasion of Si-EMC6 transfected SW1990 cells and OE-EMC6 transfected PATU-8988 cells. Cell viability and invasion of Si-APAF1 transfected ASPC1 cells and OE-APAF1 transfected PANC1 were separately evaluated using **(G,H)** the CCK8 assay and **(I)** the Transwell assay. *p ≤ 0.05, **p ≤ 0.01, ***p ≤ 0.001.

The result of the CCK8 assay showed that ATF6 overexpression and EMC6 or APAF1 knockdown enhances the growth of PC cells (Figures 5B,D,G), whereas ATF6 knockdown and EMC6 or APAF1 overexpression decreases the proliferation of PC cells compared with the control (Figures 5A,E,H). These results indicate that ATF6 promotes the viability of PC cells, while EMC6 and APAF1 have an inhibitory role.

The Transwell assay showed that the invasiveness of PC cells was markedly increased when ATF6 expression level is elevated and when EMC6 and APAF1 expression levels are reduced. However, ATF6 knockdown and EMC6 or APAF1 overexpression had the opposite effect (Figures 5C,F,I). Therefore, ATF6 promotes the invasion ability of PC cells, while EMC6 and APAF1 impair it.

## Discussion

PC has always been one of the greatest challenges of human health, especially in East Asia, where 458,918 newly diagnosed cases and approximately 432,242 death cases were recorded in 2018 (Bray et al., 2018). Despite continuing advancements in surgery, chemotherapy, radiotherapy, immunotherapy, and targeted therapy of PC, the prognosis of PC patients is still poor (Mizrahi et al., 2020; Thakur et al., 2021). Unfortunately, adjuvant and neoadjuvant treatments, which were used for PC therapy, had a scarce benefit on PC prognosis (O'Reilly and Ferrone, 2020). Thus, the evaluation of the prognosis of PC patients and the development of new therapeutic methods are in great need to be improved .

ATF6, XBP1, and CHOP were identified as core proteins in UPR signaling, which contribute to various physiological processes and cancer development (Hetz et al., 2020). The ER stress was linked to a variety of cancers and was associated with their prognosis (Nikesitch et al., 2016). Furthermore, blocking moderate ER stress and UPR could lead to tumoricidal effects (Mohamed et al., 2017). In the present study, we showed that ATF6, EMC6, XBP1, and CHOP expression are significantly higher in PC tissues compared with those in adjacent normal pancreatic tissues, indicating the potential involvement of ER stress-related proteins in PC progression. Thus, ER stress and UPR could be potential regulators of treatment effects in PC.

ATF6 is a UPR sensor that is located in the ER membrane, and that is associated with poor prognosis in Biliopancreatic and colon cancers (Martinez-Useros et al., 2015; Liu et al., 2018). Mutations in p53 result in tumor-cell differentiation and transition to malignant lesions in human PC (Morris et al., 2019). It was reported that p53 mutants enhance tumor aggressiveness by promoting cell invasion, metastasis, and chemoresistance through their interactions with ATF6 (Sicari et al., 2019). We supposed that p53 may be an ATF6 potential downstream molecule associated with a poor PC prognosis. Additionally, some studies reported that OTUB1 promotes the progression of bladder cancer through its interaction with ATF6 (Zhang et al., 2021), and that ATF6 could facilitate cervical cancer cell growth and migration through the MAPK pathway (Liu et al., 2020), resulting in poor cancer prognosis. As observed in other cancers, our study indicated that of ATF6 increased expression correlates with poor prognosis of PC. We found that the survival rate of patients was lower, and that the proliferation and aggressiveness of tumor cells were stronger in PC when ATF6 expression is elevated. Further studies are required to reveal the exact mechanism of ATF6 in cancer. These can provide opportunities for the development of new targeting therapies for PC.

EMC6 is an autophagy-related protein that is overexpressed in U2OS osteosarcoma and HCT116 colon carcinoma cells, and that participates in the formation of autophagosomes and in accelerating the degradation of autophagic substrates in lysosomes (Li et al., 2019). Meanwhile, EMC6 functions as a tumor suppressor and its overexpression induces apoptosis and cell cycle arrest in gastric cancer cells (Wang et al., 2017). Similarly, in our study, EMC6 was expressed at low levels in PC tissues from better surviving patients. EMC6 protein reduced PC cells viability and invasion, and cancer patients with high EMC6 expression had longer OS and RFS. EMC6 participates in cell autophagy through its interaction with RAB5A, and its deficiency induces the impairment of autophagy (Li et al., 2013). Recent studies showed that autophagy plays a dual role in PC progression, which suggest its potential therapeutic targeting. Facilitating and inhibiting autophagy were both effective therapeutic methods (Li et al., 2020; Piffoux et al., 2021). However, the specific mechanism of the dual effects of autophagy in PC progression is unclear, and therefore, further studies are required. In this study, EMC6 is a regulator of autophagy that exhibited an inhibitory effect on PC cells. Thus, the regulation of EMC6 expression could offer a novel direction for PC treatment.

XBP1 is considered as a biomarker of poor clinical outcomes in patients with pulmonary adenocarcinoma (Kwon et al., 2018), breast cancers (Chen et al., 2014), and multiple myeloma (Bagratuni et al., 2010). CHOP serves as an apoptosis specific transcription factor (Zhang et al., 2012), which expression is related to mesothelioma stratification of patients (Dalton et al., 2013) and cancer staging (Lee et al., 2013). In this study, we demonstrated that XBP1 and CHOP high expression occur in PC tissues, however, the correlation between XBP1 and CHOP expression and the survival of PC patients was not statistically significant.

APAF1 is as a key regulator of cell death and cell recovery pathways, and therefore, the dysregulation of apoptosis is at the root of various diseases (Gortat et al., 2015). Moreover, APAF1 expression was significantly suppressed by miR-23a in PC cells, which promotes PC cell proliferation and represses apoptosis (Liu et al., 2015). Our results revealed that APAF1 was overexpressed in PC tissues and inhibited the proliferation and invasion of PC cells, that contributed to a promising prognosis in PC patients. Shiraishi et al. reported that APAF1 plays a crucial role in ER stress-induced apoptosis (Shiraishi et al., 2006), and EMC6 has been demonstrated to influence the development of ER stress (Chitwood and Hegde, 2019). In this study, we show that APAF1 also inhibits the viability and invasion of PC cells. The exact mechanism of the interaction between ER stress and EMC6 or APAF1 on the prognosis of PC is worthy of further investigation.

## Conclusion

The expressions of ATF6, CHOP, XBP1, EMC6, and APAF1 are significantly involved in PC progression, and ATF6 overexpression and the inhibition of EMC6 or APAF1 expression are associated with poor clinical outcome in PC patients. These results suggest the potential use of these biomarkers as prognostic predictors for PC patients following surgery.

## Data Availability

The original contributions presented in the study are included in the article/Supplementary Material, further inquiries can be directed to the corresponding authors.
