# Supplementary material for: Roles and Clinical Significances of ATF6, EMC6, and APAF1 in Prognosis of Pancreatic Cancer
Source: Front Genet. 2022 Feb 11;12:730847. doi: 10.3389/fgene.2021.730847 (PMC8873166; doi:10.3389/fgene.2021.730847)
Supplement: Supplementary file 1 [file Table1.DOCX]

**Table S1. Clinical characteristic data of patients with PC**

| No. | Age | Gender | Diagnosis | Place of treatment |
| --- | --- | --- | --- | --- |
| 01 | 31-35 | female | Adenocarcinoma | Nanfang Hospital |
| 02 | 36-40 | female | Other | Nanfang Hospital |
| 03 | 36-40 | male | Adenocarcinoma | Nanfang Hospital |
| 04 | 36-40 | female | Adenocarcinoma | Nanfang Hospital |
| 05 | 36-40 | female | Adenocarcinoma | Nanfang Hospital |
| 06 | 36-40 | male | Adenocarcinoma | Nanfang Hospital |
| 07 | 36-40 | female | Adenocarcinoma | Nanfang Hospital |
| 08 | 41-45 | male | Adenocarcinoma | Nanfang Hospital |
| 09 | 41-45 | female | Other | Nanfang Hospital |
| 10 | 41-45 | male | Adenocarcinoma | Nanfang Hospital |
| 11 | 46-50 | male | Adenocarcinoma | Nanfang Hospital |
| 12 | 46-50 | female | Other | Nanfang Hospital |
| 13 | 46-50 | male | Adenocarcinoma | Nanfang Hospital |
| 14 | 46-50 | male | Adenocarcinoma | Nanfang Hospital |
| 15 | 46-50 | male | Adenocarcinoma | Nanfang Hospital |
| 16 | 46-50 | male | Adenocarcinoma | Nanfang Hospital |
| 17 | 46-50 | female | Adenocarcinoma | Nanfang Hospital |
| 18 | 46-50 | male | Adenocarcinoma | Nanfang Hospital |
| 19 | 46-50 | male | Adenocarcinoma | Nanfang Hospital |
| 20 | 51-55 | male | Adenocarcinoma | Nanfang Hospital |
| 21 | 51-55 | male | Adenocarcinoma | Nanfang Hospital |
| 22 | 51-55 | male | Adenocarcinoma | Nanfang Hospital |
| 23 | 51-55 | male | Adenocarcinoma | Nanfang Hospital |
| 24 | 51-55 | female | Adenocarcinoma | Nanfang Hospital |
| 25 | 51-55 | female | Adenocarcinoma | Nanfang Hospital |
| 26 | 51-55 | male | Adenocarcinoma | Nanfang Hospital |
| 27 | 51-55 | female | Adenocarcinoma | Nanfang Hospital |
| 28 | 51-55 | male | Adenocarcinoma | Nanfang Hospital |
| 29 | 51-55 | female | Adenocarcinoma | Nanfang Hospital |
| 30 | 51-55 | male | Adenocarcinoma | Nanfang Hospital |
| 31 | 51-55 | male | Adenocarcinoma | Nanfang Hospital |
| 32 | 51-55 | male | Adenocarcinoma | Nanfang Hospital |
| 33 | 56-60 | male | Adenocarcinoma | Nanfang Hospital |
| 34 | 56-60 | female | Adenocarcinoma | Nanfang Hospital |
| 35 | 56-60 | female | Adenocarcinoma | Nanfang Hospital |
| 36 | 56-60 | female | Adenocarcinoma | Nanfang Hospital |
| 37 | 56-60 | male | Adenocarcinoma | Nanfang Hospital |
| 38 | 56-60 | male | Adenocarcinoma | Nanfang Hospital |
| 39 | 56-60 | male | Adenocarcinoma | Nanfang Hospital |
| 40 | 56-60 | male | Adenocarcinoma | Nanfang Hospital |
| 41 | 56-60 | male | Adenocarcinoma | Nanfang Hospital |
| 42 | 56-60 | female | Adenocarcinoma | Nanfang Hospital |
| No. | Age | Gender | Diagnosis | Place of treatment |
| 43 | 61-65 | female | Adenocarcinoma | Nanfang Hospital |
| 44 | 61-65 | female | Adenocarcinoma | Nanfang Hospital |
| 45 | 61-65 | male | Adenocarcinoma | Nanfang Hospital |
| 46 | 61-65 | male | Adenocarcinoma | Nanfang Hospital |
| 47 | 61-65 | female | Adenocarcinoma | Nanfang Hospital |
| 48 | 61-65 | male | Adenocarcinoma | Nanfang Hospital |
| 49 | 66-70 | female | Adenocarcinoma | Nanfang Hospital |
| 50 | 66-70 | male | Adenocarcinoma | Nanfang Hospital |
| 51 | 66-70 | female | Adenocarcinoma | Nanfang Hospital |
| 52 | 66-70 | male | Adenocarcinoma | Nanfang Hospital |
| 53 | 66-70 | female | Adenocarcinoma | Nanfang Hospital |
| 54 | 66-70 | female | Adenocarcinoma | Nanfang Hospital |
| 55 | 66-70 | male | Adenocarcinoma | Nanfang Hospital |
| 56 | 66-70 | male | Adenocarcinoma | Nanfang Hospital |
| 57 | 66-70 | female | Adenocarcinoma | Nanfang Hospital |
| 58 | 66-70 | male | Adenocarcinoma | Nanfang Hospital |
| 59 | 66-70 | male | Adenocarcinoma | Nanfang Hospital |
| 60 | 66-70 | female | Adenocarcinoma | Nanfang Hospital |
| 61 | 71-75 | male | Adenocarcinoma | Nanfang Hospital |
| 62 | 71-75 | female | Adenocarcinoma | Nanfang Hospital |
| 63 | 71-75 | female | Adenocarcinoma | Nanfang Hospital |
| 64 | 71-75 | male | Adenocarcinoma | Nanfang Hospital |
| 65 | 71-75 | male | Adenocarcinoma | Nanfang Hospital |
| 66 | 71-75 | male | Adenocarcinoma | Nanfang Hospital |
| 67 | 76-80 | male | Adenocarcinoma | Nanfang Hospital |
| 68 | 76-80 | female | Adenocarcinoma | Nanfang Hospital |
| 69 | 76-80 | female | Adenocarcinoma | Nanfang Hospital |
